# Factors associated with academic performance among medical students at a medical school in South Korea: A retrospective cohort study

**DOI:** 10.1371/journal.pone.0296682

**Published:** 2024-02-09

**Authors:** Eun-Kyung Chung, Heoncheol Yun, Jung-Ho Yang, Min-Ho Shin, Eui-Ryoung Han

**Affiliations:** 1 Department of Medical Education, Chonnam National University Medical School, Gwangju, South Korea; 2 Institutional Research Center, Chosun University, Gwangju, South Korea; 3 Department of Preventive Medicine, Chonnam National University, Gwangju, South Korea; Alexandria University Faculty of Nursing, EGYPT

## Abstract

Longitudinal research has provided systematic empirical data on the short- and long-term outcomes of admissions policies, curricular innovations, and complex decisions on students’ academic progress. This study aimed to investigate the academic performance of medical students and related factors using cohort database collected from a medical school. The study participants included 134 medical students who graduated from Chonnam National University Medical School in 2022. The medical school’s cohort database was used to collect data on demographics, admission, academic performance, extracurricular activities, and performance on the National Korean Medical Licensing Examination (KMLE). Participating in club activities had a significant association with medical students’ academic advancement delay or leave of absence during the entire course of medical school (P = 0.007). Logistic regression analysis indicated that the nationwide clinical knowledge mock examination during the fourth year of medical school was significantly associated with passing the KMLE (adjusted odds ratio 1.12, 95% confidence interval 1.02–1.22; P = 0.014). Extracurricular school activities (a non-cognitive student attribute) and a wide range of cognitive student attributes captured from the cohort database were associated with medical students’ academic performance. In conclusion, this study can reinforce a strong emphasis on the inclusion of cognitive and non-cognitive information in medical school curricula and assessments in order to improve medical education programs and future postgraduate performance.

## Introduction

The goal of medical education is to produce competent physicians with cognitive and critical thinking skills as well as interpersonal skills in response to scientific advances and societal needs [1, 2]. To accomplish the desired outcome of producing well-rounded physicians, the paradigm of medical education has shifted to competency-based education and outcome evaluation [2]. Existing outcomes of medical school activities relevant to individual student competencies from admission to graduation can be useful to better understand how to develop competent, compassionate physicians [3]. Longitudinal databases in medical education are essential to understand how behavioral competence develop in order to set appropriate standards, and this requires following students over the full course of their educational activities [4].

Despite the importance of non-cognitive factors—defined as soft skills related to motivation, attitude, and temperament—in shaping the development of empathetic and compassionate physicians, medical curricula often lack strategies to cultivate these essential qualities [5, 6]. Medical schools often conduct longitudinal research on specific cohorts of medical students to gain a comprehensive understanding of their academic and clinical performance, as well as professional behavior, throughout the medical educational journey [7]. Most related longitudinal studies have found academic abilities (such as preclinical and clerkship grade point average [GPA], written and clinical examination scores) in medical school could predict future professional performance [8–11]. Non-cognitive factors, such as personality and behavioral qualities, have also been as significant contributors to postgraduate clinical competence [7, 12]. However, the findings were inconsistent regarding the associations between non-cognitive factors and academic outcomes [5]. Perceived stress, conscientiousness, and academic resilience were not associated with academic performance [5, 13–15]. Farrington et al. emphasized academic performance was a complex phenomenon in which cognitive and non-cognitive factors continuously interacted to create learning [6]. Therefore, it is necessary to determine association between predictor variables (including cognitive and non-cognitive student attribute) and criterion variables measured during or at the end of the course reflecting academic and clinical performance. While existing cohort studies have investigated cognitive and non-cognitive factors as predictors of academic performance in medical school [7, 16], there remains insufficient literature on this topic to draw comprehensive conclusions.

This study aimed to investigate the academic achievement of medical students and related factors using cohort database collected from a medical school. The research questions were as follows:

1) Which factors are associated with academic achievement indicators, such as academic advancement delay and leave of absence during the entire course of medical school?

2) Which factors affect final outcomes at the time of medical school graduation in terms of passing or failing the Korean Medical Licensing Examination (KMLE)?

## Methods

### Study design

A retrospective cohort study was conducted on 134 graduates who consented to have their academic performance and related factors investigated from admission to graduation. This study used data from the cohort database at Chonnam National University Medical School, which is a collection of routinely captured educational data spanning the continuum from undergraduate medical education (UME, medical school leading up to the Medical Doctor degree) to graduate medical education (GME, residency training, also known as postgraduate education, leading to eligibility for specialty board certification).

### Study participants

The study participants were a total of 134 medical students who graduated from Chonnam National University Medical School in 2022. The study was approved by the Chonnam National University Institutional Review Board (IRB No. CNUH-2022-404). All participants received a detailed explanation about their participation in the study and provided written consent to the use of their data.

### Data collection

Available UME-enrollee data included demographics (e.g., age, gender, residential area), admissions data (e.g., admission type, College Scholastic Ability Test [CSAT] grades), extracurricular activity data (e.g., club activities, student research program), academic performance data (e.g., GPA, academic advancement delay or leave of absence, nationwide medical knowledge mock examination score, regional clinical skills assessment score).

Admission data were collected from admission type and CSAT grades. Admission type was classified as regular, rolling, or transfer. In the regular admissions process, students were selected based on the results of the CSAT; for rolling admissions, students were selected based on high school grades and achieving the minimum academic ability standard on the CSAT; and for transfer admissions, students were selected based on the medical education eligibility test and university grades. Students in the area where the school is located are likely to apply for the rolling admissions or transfer programs, where school grades are more important than CSAT scores. The CSAT is divided into 9 grades. The top 4% of the standard scores are grade 1, 4–11% are grade 2, 11–23% are grade 3, 23–40% are grade 4, 40–60% are grade 5, 60–77% are grade 6, 77–89% are grade 7, 89–96% are grade 8, and 96–100% are grade 9.

Extracurricular activity data included club activities and participation in student research programs. There were a total of 30 clubs, including 15 for hobbies such as music, photography, literature, sports, and dance; 10 that promote friendship between seniors and juniors; and 5 for volunteer or religious activities. Students are free to join any number of clubs during the premedical course. Those who did not join a club during the premedical course or who stopped participating in clubs were classified as ‘no’. Student research programs allow students to participate in experimental research under the supervision of a professor in the department of basic or clinical medicine through our Medical Science Research Institute, or to be selected for an overseas basic clinical practice program and participate in experimental research under the guidance of an instructor from another medical institution during a vacation period.

Academic performance data were measured by GPA, academic advancement delay or leave of absence, and examination scores. GPA is calculated on a 4.5-point scale, and when converted to 100 points, 4.5 points (A+) equate to 95–100 points, and 1.0 point (D0) equates to 60–64 points. ‘Academic advancement delay’ refers to a student’s failure to advance to the next academic level according to the regulations—if he or she achieves either a GPA below 2.0 (C0) on the 4.5-point scale or any ‘F’ grades. The student will be withdrawn if he or she fails to meet academic advancement criteria on three occasions. ‘Leave of absence’ refers to a suspension of studies due to poor academic performance, illness, or other personal reasons. Many medical students take a leave of absence when they are unable to adapt to the high academic load or if they anticipate that they will not be able to meet the academic advancement criteria [17, 18].

Available GME-enrollee data included national exams (e.g., KMLE, Clinical Knowledge Assessment [CKA], Clinical Skills Assessment [CSA]). The national passing rate for graduates on the KMLE is approximately 94–95% per year.

We tried to obtain measurements for all variables used in this study to avoid information bias, and measurements of all variables were collected.

### Statistical analysis

Only psychological tests with many missing values were excluded; all other variables were used in the analysis. We used Student’s t-tests and chi-square tests to determine factors associated with academic advancement delay or leave of absence, as well as factors associated with passing the KMLE. Logistic regression analysis was used to investigate factors associated with passing the KMLE. All analyses were performed using SPSS Statistics for Windows, version 26.0 (IBM Corp., Armonk, NY, USA).

## Results

### Descriptive statistics

Eighty-five (63.4%) male students and 49 (36.6%) female students were included in the analysis. There were 89 (66.4%) students who resided in the area in where the school is located. Regarding the college entrance process, 45 (39.1%) students qualified through point-based regular admission. Overall, the students received a high average grade of 1.1 in the CSAT. Most students (93.3%) were participating in club activities, and only ten (7.5%) students were in student research programs (Table 1).

**Table 1:** General characteristics of fourth year medical students (N = 134).

Variable	Classification	Value, n (%) or mean ± SD
Gender	Male	85 (63.4)
	Female	49 (36.6)
Residential area	Area where the school is located	89 (66.4)
	Others	45 (33.6)
Admission type	Regular admission	45 (39.1)
	Rolling	31 (27.0)
	Transfer	39 (33.9)
College Scholastic Ability Test grades^a^ (Language Arts, Mathematics, English)		1.1 ± 0.2
Club activities	Yes	125 (93.3)
	No	9 (6.7)
Student research program	Yes	10 (7.5)
	No	124 (92.5)

^a^Presented on a scale from 1 to 9. The top 4% of the standard scores are grade 1, 4%-11% are grade 2, 11%-23% are grade 3, 23%-40% are grade 4, 40%-60% are grade 5, 60%-77% are grade 6, 77%-89% are grade 7, 89%-96% are grade 8, and 96%-100% are grade 9. SD, standard deviation

### Academic achievement during medical school

The medical students achieved mean GPA of 3.1 ± 0.7 in the 2-year premedical course and a mean GPA of 3.3 ± 0.5 in the 4-year medical course. Overall, 19 (14.2%) students experienced academic advancement delay or leave of absence during the medical school program. The students achieved a mean T-score of 114 ± 18.1 on the nationwide basic medicine mock examination during the first year of medical school and a mean T-score of 100 ± 16.0 on the nationwide clinical knowledge mock examination during the fourth year of medical school. Additionally, the students achieved a mean T-score of 50 ± 10.0 on the regional clinical skills assessment. Of the 133 students, 122 (91.7%) passed the KMLE CKA and KMLE CSA. There were seven (5.3%) and five (3.8%) students who failed the KMLE CKA and KMLE CSA, respectively (Table 2).

**Table 2:** Academic achievement during medical school.

Variable	Classification	Value, n (%) or mean ± SD
GPA during the premedical course		3.1 ± 0.7
GPA during the first year of medical school		3.3 ± 0.7
GPA during the second year of medical school		3.2 ± 0.6
GPA during the third year of medical school		3.6 ± 0.5
GPA during the fourth year of medical school		3.3 ± 0.6
GPA throughout medical course		3.3 ± 0.5
Academic advancement delay or leave of absence in the medical course	Yes	19 (14.2)
	No	115 (85.8)
Nationwide basic medicine mock examination during the first year of medical school^a^ (T-score)		114.0 ± 18.1
Nationwide clinical knowledge mock examination during the fourth year of medical school^a^ (T-score)		100.0 ± 16.0
Regional clinical skills assessment during the fourth year of medical school^b^ (T-score)		50.0 ± 10.0
KMLE CKA and CSA	Pass	122 (91.7)
	Fail	11 (8.3)
KMLE CKA	Pass	126 (94.7)
	Fail	7 (5.3)
KMLE CSA	Pass	128 (96.2)
	Fail	5 (3.8)

^a^Nationwide basic medicine and clinical knowledge mock examinations are governed by the Medical Education Assessment Consortium of the Korean Association of Medical Colleges.

^b^Regional clinical knowledge mock examination is organised by a five-medical school consortium. The third- and fourth-year clinical skills mock assessments are organised by Jeolla Consortium for Standardized Patient Education. GPA, grade point average; KMLE, Korean Medical Licensing Examination; CKA, Clinical Knowledge Assessment; CSA, Clinical Skills Assessment; SD, standard deviation

### Factors associated with medical students’ failure experiences

Participating in club activities had a significant association with medical students’ academic advancement delay or leave of absence (P = 0.007). Compared with 15(12.0%) of 125 students who participated in club activities, four (44.4%) of 9 students who did not participate in club activities experienced academic advancement delay or leave of absence. On the other hand, other variables, such as gender, residential area, and student research program participation, had no significant association with academic advancement delay or leave of absence (Table 3).

**Table 3:** Academic advancement delay or leave of absence according to the general characteristics of medical students.

Variable	No (n = 115)	Yes (n = 19)	P-value
Gender			0.979
Male	73 (85.9)	12 (14.1)	
Female	42 (85.7)	7 (14.3)	
Residential area			0.745
Area where the school is located	77 (86.5)	12 (13.5)	
Others	38 (84.4)	7 (13.6)	
Club activities			0.007
Yes	110 (88.0)	15 (12.0)	
No	5 (55.6)	4 (44.4)	
Student research program			0.181
Yes	10 (100.0)	0 (0.0)	
No	105 (84.7)	19 (15.3)	

Data are presented as n (%).

### Factors associated with passing the KMLE

Overall, high medical school GPA (P < 0.001), experience of academic advancement delay or leave of absence (P = 0.021), high nationwide basic medicine mock examination score (P = 0.028), high nationwide clinical knowledge mock examination score (P < 0.001), and high regional clinical skills assessment score (P < 0.001) were significantly associated with passing the KMLE. Students who passed the KMLE achieved higher GPA scores (mean 3.4 ± 0.5) than those who did not pass the KMLE (mean 2.8 ± 0.3). Of 115 students who did not experience academic advancement delay or leave of absence, 108 (93.9%) passed the KMLE, whereas 14 (77.8%) of 18 students who experienced academic advancement delay or leave of absence passed the final licensure examination. Students with higher scores on the nationwide basic medicine mock examination during the first year of medical school (mean 115 ± 18.1) were more likely to pass the KMLE than those with lower scores (mean 102.5 ± 14.9). Students who achieved higher scores on the nationwide clinical knowledge mock examination of the fourth year of medical school (mean 102.2 ± 14.6) were more likely to pass the KMLE than those who achieved lower scores (mean 77.4 ± 12.2). Finally, students who performed well on the regional clinical skills assessment of the fourth year of medical school (mean 115.0 ± 18.1) were more likely to pass the KMLE than those who did not perform well (mean 102.5 ± 14.9) (Table 4).

**Table 4:** Factors associated with passing the Korean Medical Licensing Examination.

Variable	Pass (n = 122)	Fail (n = 11)	P-value
Gender			0.046
Male	74 (88.1)	10 (11.9)	
Female	48 (98.0)	1 (2.0)	
Residential area			0.669
Area where the school is located	81 (91.0)	8 (9.0)	
Others	41 (93.2)	3 (6.8)	
Club activities			0.076
Yes	116 (92.8)	9 (7.2)	
No	6 (75.0)	2 (25.0)	
Student research program			0.323
Yes	10 (100.0)	0 (0.0)	
No	112 (91.1)	11 (8.9)	
GPA of the medical course	3.4 ± 0.5	2.8 ± 0.3	<0.001
Academic advancement delay or leave of absence in the medical course			0.021
Yes	14 (77.8)	4 (22.2)	
No	108 (93.9)	7 (6.1)	
Nationwide basic medicine mock examination during first year of medical school (T-score)	115.0 ± 18.1	102.5 ± 14.9	0.028
Nationwide clinical knowledge mock examination during the fourth year of medical school (T-score)	102.2 ± 14.6	77.4 ± 12.2	<0.001
Regional clinical skills assessment during the fourth year of medical school (T-score)	51.0 ± 9.4	39.4 ± 11.2	<0.001

Data are presented as n (%) or mean ± standard deviation. GPA, grade point average

Logistic regression analysis indicated that the nationwide clinical knowledge mock examination during the fourth year of medical school was significantly associated with passing the KMLE (adjusted odds ratio 1.12, 95% confidence interval 1.02–1.22; P = 0.014; Table 5).

**Table 5:** Logistic regression analysis of the factors associated with passing the Korean Medical Licensing Examination.

Variable	Adjusted OR (95% CI)	P-value
Gender (ref. Male)		
Female	1.44 (0.11–19.28)	0.784
Residential area (ref. Others)		
Area where the school is located	0.95 (0.16–5.61)	0.952
Club activities (ref. No)		
Yes	2.89 (0.14–61.34)	0.496
GPA throughout medical course	13.33 (0.34–517.54)	0.165
Academic advancement delay or leave of absence in the medical course (ref. No)		
Yes	0.89 (0.14–5.64)	0.900
Nationwide basic medicine mock examination during first year of medical school (T-score)	0.98 (0.92–1.05)	0.635
Nationwide clinical knowledge mock examination during the fourth year of medical school (T-score)	1.12 (1.02–1.22)	0.014
Regional clinical skills assessment during the fourth year of medical school (T-score)	1.07 (0.98–1.18)	0.144

OR, odds ratio; CI, confidence interval; GPA, grade point average

## Discussion

This study investigated the academic achievement of medical students and associated factors using cohort data collected from a medical school database.

The contribution of the current study is twofold. First, we found that students’ participation in extracurricular school activities (a non-cognitive student attribute) is significantly associated with academic advancement delay or a leave of absence from the medical school. Specifically, this study extends insight into the importance of persistent participation in extracurricular activities during medical school, which is essential for preventing detrimental academic consequences, such as academic advancement delay or a leave of absence and promoting positive academic outcomes, such as high GPAs [19, 20]. Second, this study revealed that a wide range of assessment measures (cognitive student attributes, e.g., GPA, academic advancement delay, and nationwide medical examination scores) obtained from the existing cohort database within our institution were strongly associated with performance on the KMLE. The findings from this study demonstrate that, to deepen our understandings of students’ performance and the effectiveness of medical education, medical educators and researchers can benefit from using a combination of measures from existing sources [3, 7].

Our study found that consistent involvement in extracurricular activities during medical school was a significant facilitator of improved learning outcomes and academic performance among medical students. This finding aligns with prior studies that have demonstrated extracurricular activities to have a significant impact on quality of life as well as academic performance among medical students [20–22]. Extracurricular activities refer to non–credit-granting and voluntary activities that are carried out by students outside of the medical school curriculum; these can include research, teaching, community, cultural, social, religious, and sporting activities [23]. Medical educators have highly prioritized medical students’ quality of life because it may impact their academic performance and vice versa [21]. The fear of academic advancement delay is the major stressor during medical school, and it can negatively impact medical students’ quality of life [22]. Medical students participate in a variety of academic and extracurricular activities in order to improve their work-life balance [21]. On the other hand, some research has shown that medical institutions tend to heavily focus on extracurricular research programs to enhance research competency because medical students have strong interest in developing professionalism through extracurricular research participation [24]. Conversely, this may lead to negative outcomes, such as stress and burnout [25]. Redundant participation in extracurricular activities can also result in failure in academic examinations [26]. Hence, based on our findings, we contend that extracurricular activities should not only mitigate the stress associated with the fear of academic advancement delay but also improve the overall well-being of medical students. To encourage students to actively participate in extracurricular activities, medical schools should be aware of potential motives for further engagement and remove obstacles that disrupt participation; concurrently, the implementation of faculty mentorship, activity evaluation systems, and career association programs should take place for the further development of extracurricular activities [23, 27].

The evidence from this study suggests that cognitive student attributes related to academic performance during medical school may be predictors of performance on the KMLE and may reflect the strengths and weaknesses of medical education curricula. Specifically, the KMLE, with which prospective physicians are expected to demonstrate professional clinical knowledge and skills, is a profound assessment of the effectiveness of undergraduate medical education programs and a critical predictor of performance during their postgraduate training [8]. In this regard, this study supports the findings from previous studies that pre-clinical performance and exam preparation are significantly associated with performance on medical licensure examinations, such as USMLE (United States Medical Licensing Examination) Step 1 [28, 29]. Likewise, medical students with higher scores in academic and clinical examinations were more likely to demonstrate better outcomes in the KMLE in our study. We may subsequently develop a predictive assessment of clinical competence for physicians in postgraduate professional programs based on such cohort data [3, 28]. However, this research should look beyond relationships between exam performance and associated factors that can be derived from a cohort database. For example, relevant research noted that medical students’ exam-directed study behaviors are positively predictive of high-stakes examination performance [29], and institutional factors such as coaching and help-seeking can be feasible approaches for students at risk of exam failure [30].

This study had several limitations. First, the findings inherently cannot be generalized to other medical education contexts because this study used an existing cohort database from specific medical students at one medical school. Second, we recommend that future studies include a broad range of non-cognitive student attributes such as individual characteristics (e.g., personal traits, interpersonal skills, and self-appraisal), tutor evaluations, and socioeconomic factors, in order to gain a deeper understanding of predictors of academic advancement delay and performance on a medical licensure examination [7, 12]. Third, potential unmeasured confounding factors could affect the interpretation of academic performance and related factors in medical school. For instance, variations in academic ability may arise from different admission types. Reasons for a leave of absence may extend beyond poor academic performance, such as personal or health-related issues. Academic challenges may also impede participation in extracurricular activities. Furthermore, the COVID-19 pandemic could have impacted academic performance through factors like inadequate support, economic hardship, and individual health challenges. Finally, this study findings suggest that medical educators and researchers should have access to the data of individualized learners and use the predictive techniques of learning analytics (LA) to offer early interventions to at-risk medical students because we focused on assessing the relationships between predictors and academic performance in medical examinations [3, 31].

## Conclusions

Undergraduate medical students’ participation in extracurricular activities was significantly associated with academic advancement delay or a leave of absence from the medical school. A variety of assessment measures obtained from our medical school’s existing cohort database were highly associated with student performance on the KMLE. In conclusion, this study can reinforce a strong emphasis on the inclusion of cognitive and non-cognitive information in medical school curricula and assessments in order to improve medical education programs and future postgraduate performance.

## References

[1] Buja LM. Medical education today: all that glitters is not gold. BMC Med Educ. 2019;19(1):1–11. 30991988 10.1186/s12909-019-1535-9 PMC6469033

[2] Carraccio C, Wolfsthal SD, Englander R, Ferentz K, Martin C. Shifting paradigms: from Flexner to competencies. Acad Med. 2002;77(5):361–7. doi: 10.1097/00001888-200205000-00003 12010689

[3] Cook DA, Andriole DA, Durning SJ, Roberts NK, Triola MM. Longitudinal research databases in medical education: facilitating the study of educational outcomes over time and across institutions. Acad Med. 2010;85(8):1340–6. doi: 10.1097/ACM.0b013e3181e5c050 20671463

[4] Krupat E. Critical thoughts about the core entrustable professional activities in undergraduate medical education. Acad Med. 2018;93(3):371–6. doi: 10.1097/ACM.0000000000001865 28857790

[5] Chisholm-Burns MA, Berg-Poppe P, Spivey CA, Karges-Brown J, Pithan A. Systematic review of noncognitive factors influence on health professions students’ academic performance. Adv Health Sci Educ. 2021;26(4):1373–445. doi: 10.1007/s10459-021-10042-1 33772422

[6] Farrington CA, Roderick M, Allensworth E, Nagaoka J, Keyes TS, Johnson DW, et al. Teaching adolescents to become learners. The role of noncognitive factors in shaping school performance: A critical literature review: University of Chicago Consortium on School Research; 2012.

[7] Adam J, Bore M, Childs R, Dunn J, Mckendree J, Munro D, et al. Predictors of professional behaviour and academic outcomes in a UK medical school: a longitudinal cohort study. Med Teach. 2015;37(9):868–80. doi: 10.3109/0142159X.2015.1009023 25665628

[8] Hamdy H, Prasad K, Anderson MB, Scherpbier A, Williams R, Zwierstra R, et al. BEME systematic review: predictive values of measurements obtained in medical schools and future performance in medical practice. Med Teach. 2006;28(2):103–16. doi: 10.1080/01421590600622723 16707291

[9] Markert RJ. The relationship of academic measures in medical school to performance after graduation. Acad Med. 1993;68(2):S31–4. doi: 10.1097/00001888-199302000-00027 8141856

[10] Part HM, Markert RJ. Predicting the first-year performances of international medical graduates in an internal medicine residency. Acad Med. 1993. 8216659

[11] Han E-R, Chung E-K. Does medical students’ clinical performance affect their actual performance during medical internship? Singapore Med J. 2016;57(2):87–91. doi: 10.11622/smedj.2015160 26768172 PMC4759381

[12] Hojat M, Borenstein BD, Veloski JJ. Cognitive and noncognitive factors in predicting the clinical performance of medical school graduates. Acad Med. 1988;63(4):323–5. doi: 10.1097/00001888-198804000-00009 3357184

[13] Gazzaz ZJ, Baig M, Al Alhendi BSM, Al Suliman MMO, Al Alhendi AS, Al-Grad MSH, et al. Perceived stress, reasons for and sources of stress among medical students at Rabigh Medical College, King Abdulaziz University, Jeddah, Saudi Arabia. BMC Med Educ. 2018;18(1):1–9. 29471824 10.1186/s12909-018-1133-2 PMC5824476

[14] Ferguson E, Semper H, Yates J, Fitzgerald JE, Skatova A, James D. The ‘dark side’and ‘bright side’of personality: When too much conscientiousness and too little anxiety are detrimental with respect to the acquisition of medical knowledge and skill. PLoS One. 2014;9(2):e88606. doi: 10.1371/journal.pone.0088606 24586353 PMC3937323

[15] Wilkinson TJ, McKenzie JM, Ali AN, Rudland J, Carter FA, Bell CJ. Identifying medical students at risk of underperformance from significant stressors. BMC Med Educ. 2016;16(1):1–9. doi: 10.1186/s12909-016-0565-9 26837428 PMC4739335

[16] Žuljević MF, Buljan I. Academic and non-academic predictors of academic performance in medical school: an exploratory cohort study. BMC Med Educ. 2022;22(1):1–9. 35562795 10.1186/s12909-022-03436-1 PMC9098375

[17] Roh M-S, Jeon HJ, Kim H, Han SK, Hahm B-J. The prevalence and impact of depression among medical students: a nationwide cross-sectional study in South Korea. Acad Med. 2010;85(8):1384–90. doi: 10.1097/ACM.0b013e3181df5e43 20453812

[18] Han ER, Chung EK, Oh SA, Chay KO, Woo YJ. Medical students’ failure experiences and their related factors. Korean J Med Educ. 2012;24(3):233–40. doi: 10.3946/kjme.2012.24.3.233 25813132 PMC8813349

[19] Ferguson E, James D, Madeley L. Factors associated with success in medical school: systematic review of the literature. BMJ. 2002;324(7343):952–7. doi: 10.1136/bmj.324.7343.952 11964342 PMC102330

[20] Urlings-Strop LC, Themmen AP, Stegers-Jager KM. The relationship between extracurricular activities assessed during selection and during medical school and performance. Adv Health Sci Educ. 2017;22(2):287–98. doi: 10.1007/s10459-016-9729-y 27812819

[21] Lumley S, Ward P, Roberts L, Mann JP. Self-reported extracurricular activity, academic success, and quality of life in UK medical students. Int J Med Educ. 2015;6:111–7. doi: 10.5116/ijme.55f8.5f04 26385285 PMC4583828

[22] Almalki SA, Almojali AI, Alothman AS, Masuadi EM, Alaqeel MK. Burnout and its association with extracurricular activities among medical students in Saudi Arabia. Int J Med Educ. 2017;8:144–50. doi: 10.5116/ijme.58e3.ca8a 28454079 PMC5420457

[23] Almasry M, Kayali Z, Alsaad R, Alhayaza G, Ahmad MS, Obeidat A, et al. Perceptions of preclinical medical students towards extracurricular activities. Int J Med Educ. 2017;8:285–9. doi: 10.5116/ijme.5973.297a 28817380 PMC5572427

[24] Kim S, Jeong H, Cho H, Yu J. Extracurricular activities in medical education: an integrative literature review. BMC Med Educ. 2023;23(1):1–11. 37087451 10.1186/s12909-023-04245-w PMC10122317

[25] Mukesh HV, Acharya V, Pillai R. Are extracurricular activities stress busters to enhance students’ well-being and academic performance? Evidence from a natural experiment. J Appl Res High Educ. 2023;15(1):152–68.

[26] Slade AN, Kies SM. The relationship between academic performance and recreation use among first-year medical students. Med Educ Online. 2015;20(1):25105. doi: 10.3402/meo.v20.25105 25819693 PMC4376935

[27] Nikkar-Esfahani A, Jamjoom AA, Fitzgerald JEF. Extracurricular participation in research and audit by medical students: opportunities, obstacles, motivation and outcomes. Med Teach. 2012;34(5):e317–e24. doi: 10.3109/0142159X.2012.670324 22471919

[28] Parry S, Pachunka J, Beck Dallaghan GL. Factors predictive of performance on USMLE step 1: do commercial study aids improve scores? Med Sci Educ. 2019;29(3):667–72. doi: 10.1007/s40670-019-00722-4 34457530 PMC8368955

[29] Burk-Rafel J, Santen SA, Purkiss J. Study behaviors and USMLE Step 1 performance: implications of a student self-directed parallel curriculum. Acad Med. 2017;92(11S):S67–S74. doi: 10.1097/ACM.0000000000001916 29065026

[30] Monrad SU, Wolff M, Kurtz J, Deiorio NM, Sabo R, Stringer JK, et al. What is the association between student well‐being and high‐stakes examination scores? Med Educ. 2021;55(7):872–7. doi: 10.1111/medu.14460 33501719

[31] Saqr M, Fors U, Tedre M. How learning analytics can early predict under-achieving students in a blended medical education course. Med Teach. 2017;39(7):757–67. doi: 10.1080/0142159X.2017.1309376 28421894

